# Beta-amyloid influences the content and trafficking of beta-amyloid precursor protein via Na,K-ATPase-Src kinase positive feedback loop

**DOI:** 10.3389/fphar.2025.1665715

**Published:** 2025-09-24

**Authors:** Irina Y. Petrushanko, Denis R. Lisitskii, Filipp A. Filonov, Olga G. Leonova, Vladimir A. Mitkevich, Maria A. Strelkova, Alexander A. Makarov

**Affiliations:** Engelhardt Institute of Molecular Biology Russian Academy of Sciences, Moscow, Russia

**Keywords:** beta-amyloid, APP, Src kinase, ouabain, APP trafficking, Na,K-ATPase

## Abstract

Beta-amyloid (Aβ) is an important factor in the development of pathology in Alzheimer’s disease. Level of beta-amyloid precursor protein (APP) is increased in neurites with age and in Alzheimer’s disease model mice. However, it is unclear whether Aβ can affect APP levels in cells. The aim of this study was to evaluate the effect of Aβ on the level and trafficking of APP in human neuroblastoma cells and to identify the role of cardiotonic steroid (CTS) ouabain in this process. Western blot analysis revealed that 30-min incubation of the cells with 100 nM Aβ increased APP levels by 75%. Confocal microscopy showed that Aβ alters APP trafficking, promoting its movement into neurites. This effect establishes a positive feedback loop that accelerates Aβ formation in neurites. The rise in APP was associated with Src kinase activation triggered by Aβ binding to Na,K-ATPase. Notably, Src kinase inhibition completely blocked the Aβ-induced increase in APP, indicating that beta-amyloid effect on APP is mediated by Src kinase activation. Furthermore, 100 nM CTS ouabain, a specific Na,K-ATPase ligand, significantly decreased Aβ′s impact on APP and Src kinase activation. Given that CTS are naturally present in the human body, these findings are important for developing therapeutic strategies to counteract Aβ-driven APP accumulation and for understanding the role of endogenous CTS in regulating Aβ formation.

## Introduction

Beta-amyloid peptide (Aβ), and mutations that lead to its accelerated oligomerization are the key to the onset of Alzheimer’s disease (AD), one of the most common neurodegenerative diseases (Prusiner, 2012; Jucker and Walker, 2013; Ma et al., 2022). This disease leads to synapse loss, neuronal dysfunction and death, causing progressive dementia accompanied by the formation of amyloid plaques in the brain (Lane et al., 2018). The risk of the emergence of AD increases with age (Burrinha and Guimas Almeida, 2022).

Aβ is produced from beta-amyloid precursor protein (APP) by β-site APP-cleaving enzyme 1 (BACE1) and gamma-secretase (Orobets and Karamyshev, 2023). Membrane trafficking of APP and BACE 1 determines the probability of their contact and consequently the rate of Aβ production (Sun and Roy, 2018). In neuronal cells, the interaction between APP and BACE1 has been detected in the cytoplasmic membrane, endoplasmic reticulum (ER), trans-Golgi network, and endosomes (Sun and Roy, 2018). APP is represented in all parts of the neuronal cell including soma, axons, dendrites, and synaptic sites (Sun and Roy, 2018). From the trans-Golgi network, APP is transported to the cytoplasmic membrane (Müller et al., 2017), where it is processed through either a non-amyloidogenic pathway, which leads to the formation of the neuroprotective soluble APP (after cleavage by α-secretase and γ-secretase) (Müller et al., 2017), or an amyloidogenic pathway, which results in Aβ formation (after cleavage by BACE1 and gamma-secretase).

Aβ is secreted pre- and postsynaptically (Niederst et al., 2015) and then binds to the post- or presynaptic membrane, being further captured to the cell by endocytosis. Accumulation and oligomerization of Aβ affects synaptic plasticity (Perdigão et al., 2020). On the membrane surface of neuronal cells, Aβ binds several proteins (Jarosz-Griffiths et al., 2016). One important target of Aβ is Na,K-ATPase (Dickey et al., 2005), which creates the sodium-potassium gradient crucial for neuronal viability and functionality. In Alzheimer’s disease, a steady decrease in Na,K-ATPase activity is observed (Dickey et al., 2005; Zhang et al., 2013; Kreutz et al., 2013). This is caused by Aβ binding (Petrushanko et al., 2016) and subsequent oligomerization on the Na,K-ATPase (Barykin et al., 2018). Particularly, we have previously shown that a monomer of Aβ acts as a ligand of Na,K-ATPase that, being bound, induces the activation of Src kinase (Petrushanko et al., 2022). Since Src kinase, in its turn, regulates APP trafficking by phosphorylating Munc-18–1 interacting protein (Mint) (Dunning et al., 2016), we hypothesized (Petrushanko et al., 2022) that Aβ may also influence the trafficking of its precursor protein by creating a positive feedback loop via Src kinase activation.

Aβ accumulation has been shown in the aging brain (Marks et al., 2017; Koinuma et al., 2021; Welikovitch et al., 2018). Additionally, it is shown that APP processing also increases with age (Burrinha et al., 2021). Presumably, this happens due to altered APP trafficking in aging neurons (Burrinha and Guimas Almeida, 2022). In murine cortical senescent neurons, disturbed APP trafficking has been shown *in vitro* and *in vivo*. As a result, APP accumulates in neurites with age (Burrinha et al., 2021). However, until now, it has not been determined whether Aβ can directly affect APP content and transport.

In the present study, we demonstrated on SY-SY5Y human neuroblastoma cells that Aβ_42_ leads to an increase in the total level of APP and its accumulation in the neurites of the cells. Since cardiotonic steroid ouabain is a specific ligand of Na,K-ATPase (Schatzmann, 1953) that is also able to induce the activation of Na,K-ATPase-associated Src kinase (for review see Orlov et al., 2021; Poluektov et al., 2025), ouabain was presumed to modulate the action of Aβ_42_ on Src kinase activation. Ouabain-like factor is present in cerebrospinal fluid (Halperín et al., 1983) and is produced directly in the brain (Bagrov et al., 2009). Acute binding of ouabain to Na,K-ATPase does not affect its binding to Aβ_42_ (Adzhubei et al., 2022), it is reasonable to suggest that this ligand may bind Na,K-ATPase simultaneously with Aβ_42_, influencing the cellular response to Aβ_42_. We characterized the effect of ouabain on Aβ_42_-induced changes in APP level and proved its ability to attenuate APP accumulation induced by Aβ_42_.

## Materials and methods

### Cell line

Human neuroblastoma cell line SH-SY5Y obtained from the American Type Culture Collection was cultured in RPMI-1640 medium (Gibco, ThermoFisher Scientific, Waltham, MA, United States), containing 10% fetal bovine serum (FBS; Gibco, ThermoFisher Scientific, MA, United States), 100 units/mL penicillin, 100 μg/mL streptomycin, pyruvate and glutamax (Gibco, ThermoFisher Scientific, Waltham, MA, United States). Culture maintenance was performed in cultural flasks T-25 and T-75 at 37 °C in humid atmosphere, containing 5% CO_2_. SH-SY5Y cells were dissociated via washing with Versene solution (Gibco, ThermoFisher Scientific, Waltham, MA, United States) and 0.05% trypsin-EDTA (Gibco, ThermoFisher Scientific, Waltham, MA, United States) digestion at 37 °C during 5 min. Passages did not exceed 15. For confocal microscopy cells were seeded on 35 mm glass-based Petri dishes (Nunc, Rochester, NY, United States, 150680) in a quantity 15000 per dish. For Western blotting, redox parameters and Ca^2+^ level measurements SH-SY5Y cells were grown on 6- and 12-well plates until 80%–90% confluency was achieved.

### Aβ_42_ preparation

Synthetic peptide Aβ_42_: [H2N]-DAEFRHDSGYEVHHQKLVFFAEDVGSNKGAIIGLMVGGVVIA-[COOH] was obtained from Biopeptide (San Diego, CA, United States). Preparation of the monomeric form of Aβ_42_ was performed as described elsewhere. Cold hexafluoroisopropanol (Fluka) was added to dry Aβ_42_ until peptide concentration 1 mM was achieved. After 1 h incubation peptide solution was transferred on ice for 10 min and aliquoted into microcentrifuge tubes (0.56 mg Аβ_42_ per tube). Aliquots were dried under vacuum using Eppendorf Concentrator 5301. Dried peptide films were stored at −80 °C. 2.5 mM stock solution was prepared by dissolving 0.22 mg of dry peptide in 20 μL of 100% anhydrous DMSO (Sigma-Aldrich, St. Louis, MO, United States) and 1-h incubation. The required concentration of Aβ_42_ was achieved by dilution of stock solution with RPMI-1640 medium or Tyrode solution. An equivalent volume of pure DMSO was added to control probes. Only fresh-dissolved Aβ_42_ was used in experiments.

### Aβ_42_ and ouabain treatment

Incubation of SH-SY5Y cells on plates or dishes with amyloid peptide required medium replacement with FBS-free RPMI-1640. For the flow cytometry studies SH-SY5Y сells were dissociated and suspended in Tyrode solution with following staining before incubations with ouabain and Aβ_42_. Aβ_42_ stock solution was diluted and added to samples in final concentration 100 nM. Beta-amyloid effects was studied by SH-SY5Y incubation for 30 min. Influence of ouabain or Src kinase inhibitor 1 (SRCI1) was estimated by pre-treatment with single compounds for 30 min before adding of Aβ_42_ solution. Ouabain (Fluka) was used in 100 nM concentration, SRCI1 – 10 μM. All incubations were performed at 37 °C in humid atmosphere, containing 5% CO_2_.

### Amyloid precursor protein distribution studies

SH-SY5Y cells were cultured on Petri dishes until 50% confluency was achieved. Medium was replaced with FBS-free RPMI-1640 and cells were incubated with 100 nM Aβ_42_ during 15–240 min. For each time point own control without addition of Aβ_42_ was performed. As incubations ended cells were washed with ice-cold Ca^2+^/Mg^2+^ PBS and fixed in 4% para-formaldehyde solution for 10 min. At the end of fixation cells were washed with Ca^2+^/Mg^2+^ PBS and treated with monoclonal rabbit antibodies against N-terminal extracellular domain of amyloid precursor protein (dilution in PBS to the concentration 3.84 μg/mL, Abcam Limited, Discovery Drive, Cambridge Biomedical Campus, Cambridge, United Kingdom, ab126732) at 4 °C overnight. Next day, samples were washed with Ca^2+^/Mg^2+^ PBS and incubated for 2 h at room temperature with secondary antibodies, conjugated with AlexaFluor^®^ 488 (Ex/Em = 495/519 nm, dilution in PBS to the concentration 4 μg/mL, Abcam Limited, Discovery Drive, Cambridge Biomedical Campus, Cambridge, United Kingdom, ab150077). Before the imaging nuclei were stained with NucBlue™ (Hoechst 33342) (Ex/Em = 360/460, Invitrogen, ThermoFisher Scientific, MA, United States, R37605) in accordance with the manufacturer’s protocol.

### Laser scanning confocal microscopy

The attached and stained SH-SY5Y cells in the 35 mm glass-based Petri dishes were covered with Ca^2+^/Mg^2+^ PBS and imaged using a confocal microscope Leica TCS SP5 (Leica, Wetzlar, Germany). APP was labeled with AlexaFluor^®^ 488-conjugated antibodies and imaged using a 488 nm argon laser. Nuclei were stained with NucBlue™ (Hoechst 33342) and visualized with 405 nm diode laser. The resulting images were analyzed using LAS X imaging software (Leica, Wetzlar, Germany). Received images were analyzed using ImageJ 1.54 g (Wayne Rasband and contributors, National Institutes of health, United States). The fluorescence ratio between neurites and cell bodies was calculated, value of every single time measurement was normalized to control with the same time of incubation.

### Estimation of APP levels and Src kinase activation

Cells were grown on 6- and 12-well plates until 80%–90% confluency was achieved. Medium was replaced with FBS-free RPMI-1640 and cells were incubated with Aβ_42_, cardiotonic steroids and Src kinase inhibitor 1. After the ending of incubation wells were washed with Ca^2+^/Mg^2+^ PBS and cells were lysed with RIPA-buffer (ThermoFisher Scientific, Waltham, MA, United States, 89900), containing protease inhibitors cocktail (Roche, 11836145001), phosphatase inhibitors cocktail (Roche, 4906837001), 0.2 mM PMSF and 5 μM thiorphan (Cayman Chemical, Ann Arbor, MI, United States, 15600), with stirring for an hour at 4 °C. Lysates were centrifuged at 4 °C and 16000 g for 10 min and supernatants were collected.

The cell lysates were separated via 10% SDS-PAGE electrophoresis and transferred to a PVDF-membrane (Bio-Rad, Hercules, CA, United States, 1620137). Membranes were blocked in 5% nonfat milk in TBST (50 mM Tis-HCl, pH 7.4, 150 mM NaCl, 0.1% Tween-20) for an hour and were incubated with monoclonal primary rabbit antibodies specific to APP (dilution in 5% milk-TBST to the concentration 76,8 ng/mL, Abcam Limited, Discovery Drive, Cambridge Biomedical Campus, Cambridge, United Kingdom, ab32136), β-actin (dilution in TBST to the concentration 60 ng/mL, Abcam Limited, Discovery Drive, Cambridge Biomedical Campus, Cambridge, United Kingdom, ab8227), Src kinase (dilution in 5% milk-TBST to the concentration 67 ng/mL, Cell Signaling Technology, Danvers, MA, United States, 2108S) and Phospho-Src Family (Tyr416) (dilution in 5% milk-TBST to the concentration 51 ng/mL, Cell Signaling Technology, Danvers, MA, United States, 6943S) overnight at 4 °C. Then, the membranes were washed in TBST and incubated with goat anti-rabbit secondary antibodies, conjugated with HRP (dilution in TBST to the concentration 140 ng/mL, HyTest, Moscow, Russia, GARC). Imaging of the membranes was performed using SuperSignal™ West Femto Maximum Sensitivity Substrate kit (ThermoFisher Scientific, MA, United States, 34096) and Bio-Rad ChemiDoc MP instrument (Bio-Rad, Hercules, CA, United States). Densitometric analysis was performed with Image Lab 6.0.1 program (Bio-Rad, Hercules, CA, United States). Results were expressed as APP levels, normalized on that in control (APP, %), or phospho-Src/Src ratio (p-Src/Src, fold change).

### Assessment of Ca^2+^ levels, mitochondrial potential and redox status of the cells

SH-SY5Y cells were grown on 12-well plates at 37 °C in humid atmosphere, containing 5% CO_2_, and suspended in Tyrode solution after achieving 80%–90% confluency. Then, suspensions from every well were divided into two parts, every sample was stained simultaneously with GSH- and Ca^2+^-specific or ROS- and mitochondrial membrane potential-specific dyes. The ROS level was assessed using the dihydrorodamine 123 (DHR) dye (Ex/Em = 488/525 nm; Invitrogen, ThermoFisher Scientific, MA, United States, D23806, used in a concentration 5 μM). Assessment of the Ca^2+^ level was performed by staining with fluo-4, AM (Ex/Em = 494/506 nm; Invitrogen, ThermoFisher Scientific, MA, United States, F14201). The monobromobimane dye (Ex/Em = 393/490 nm, Sigma-Aldrich, St. Louis, MO, B4380, used in a concentration 20 μM) was used for the reduced glutathione (GSH) staining. The mitochondrial potential was assessed using the MitoProbe™ DiIC1(5) Assay Kit (Ex/Em = 638/658 nm; Invitrogen, ThermoFisher Scientific, MA, United States, M34151, used in a concentration 50 nM). All parameters were recorded for the cells with intact membrane. The dyes were incubated at 37 °C for 30 min. Detection of the cells with damaged membrane (dead cells) was performed by staining with propidium iodide (PI) (Ex/Em = 535/617 nm, Sigma-Aldrich, St. Louis, MO, P4170, used in a concentration 10 μg/mL) 1 minute before measurement. When assessing the levels of ROS, GSH and mitochondrial potential, dead cells were excluded from consideration. The cells were analyzed using a flow cytometer BD LSR Fortessa (Becton Dickinson, Franklin Lakes, NJ, United States).

### Statistical analysis

All experimental data are shown as mean values ± standard deviations of mean (SD), with the number of independent experiments (n) indicated in Figure legends. The statistical difference between experimental groups was analyzed by one-way ANOVA with Tukey correction for multiple comparisons. Probability values (p) less than 0.05 were considered significant. Statistical analysis was performed using GraphPad Prism 9.1.2 software (GraphPad Software Inc., San Diego, CA, United States).

## Results

### Аβ_42_ increases APP level and alters its distribution in SH-SY5Y cells

**FIGURE 1:**
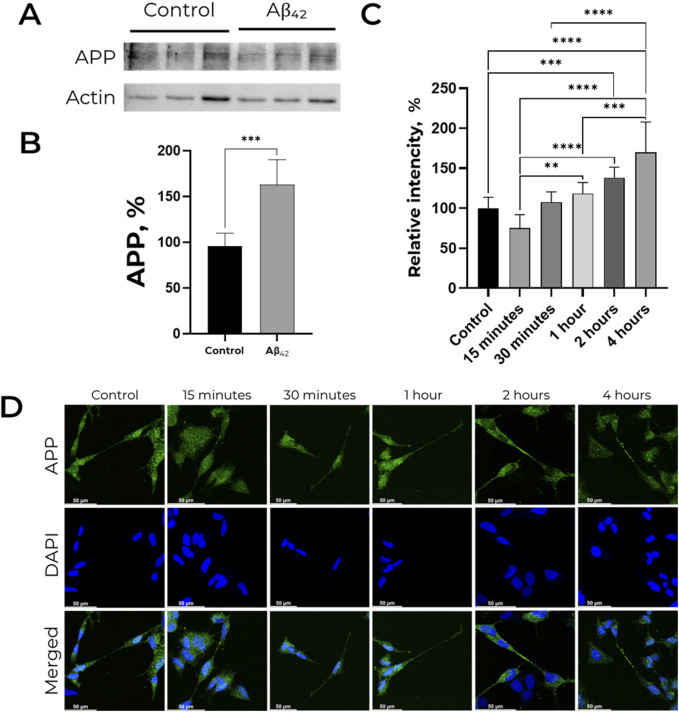
Effect of Aβ_42_ on APP expression and distribution in SH-SY5Y cells. **(A,B)** APP level is increased in presence of 100 nM Aβ_42_. For each lane, the APP (∼120 kDa) signal was normalized to actin (∼40 kDa). APP levels were measured by Western blot analysis of lysates of human neuroblastoma SH-SY5Y cells treated with 100 nM Aβ_42_ for 30 min. Full-size Western blot membranes, from which the bar plots were calculated, are provided in the Supplementary. Mean values ± SD from at least three independent experiments are shown. **(C,D)** Localization of amyloid precursor protein in SH-SY5Y cells incubated with 100 nM Aβ_42_. APP was labeled with AlexaFluor^®^ 488-conjugated antibodies and is indicated by green color, nuclei are stained with NucBlue (Hoechst 33342) and indicated by blue color. For each incubation time, the ratio of fluorescence in neurites and cell bodies was calculated, and the values were normalized to the control. Mean values ±SD, n = 3 from at least three independent fields are shown. * – p < 0.05, ** – p < 0.01, *** – p < 0.001 compared to the control.

Western blot analysis revealed that 30-min of incubation with 100 nM Аβ_42_ causes significant increase in APP level in SH-SY-5Y cells by 75% (Figures 1A,B). Using confocal microscopy, we evaluated the change of fluorescence level in neurites relative to the cell body after 15, 30 min, 1, 2 and 4 h of incubation with 100 nM Aβ_42_ (Figures 1C,D). In cells untreated with primary antibodies to APP signal was not detected (Supplementary Figure S1). Relative fluorescence in neurites after 1 hour of incubation is higher than after 15 min of incubation with Aβ_42_. After 2 h of Aβ_42_ exposure, the relative fluorescence is significantly increased compared to the control cells. After 4 h, the relative fluorescence in neurites already exceeds this value in the control cells by 1.5 times. All in all, Аβ_42_ induces the increase in APP level and its latter accumulation on the surface of neurites.

### Src kinase mediates Aβ_42_-dependent APP accumulation in a redox-independent way

**FIGURE 2:**
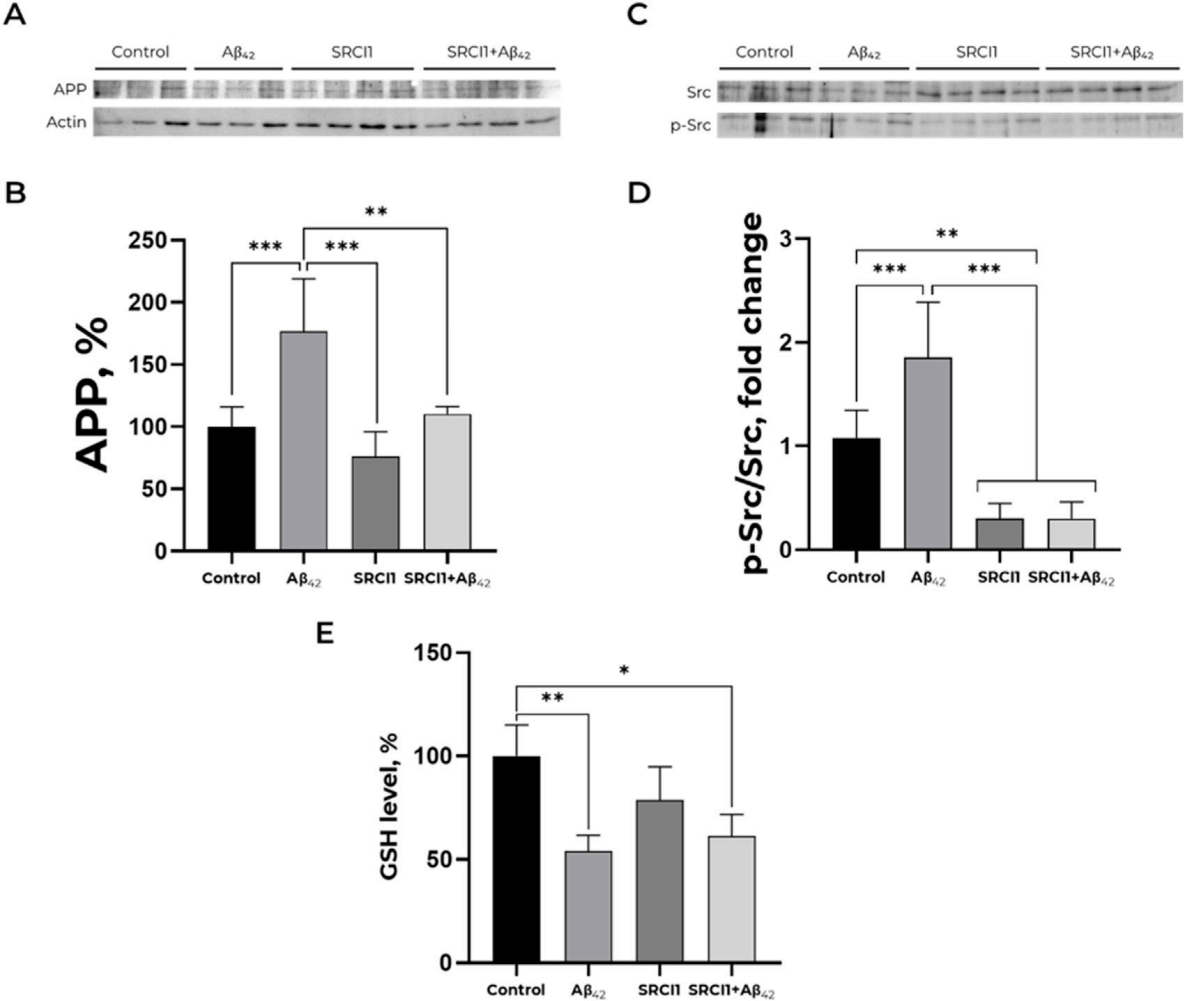
Inhibition of Src by SRCI1 prevents amyloid-mediated increase of APP level but does not affect GSH level decrease induced by Aβ_42_. **(A,B)** Impact of Aβ_42_, SRCI1 and Aβ_42_ after SRCI1 pre-treatment on APP expression. The APP (∼120 kDa) to actin (∼40 kDa) ratio has been calculated for every single band. Mean values ± SD from at least three independent experiments are shown. **(C,D)** Dependence of Src kinase activation on the presence of Aβ_42_ and/or SRCI1. The ratio of phospho (Tyr)-416 Src to the total Src (∼60 kDa) has been calculated. The APP, phosphorylated and total Src levels were measured with Western blot in SH-SY5Y human neuroblastoma cells treated with 100 nM Aβ_42_, 10 µM SRCI1 or both for 30 min and normalized for control. Mean values ± SD, n = 3-4 are shown. **(E)** Reduced glutathione alteration in a presence of Aβ_42_, Src kinase inhibitor 1 and both. The SH-SY5Y human neuroblastoma cells were harvested and stained with monobromobimane for GSH measurements and incubated with 100 nM Aβ_42_ and, if required, 10 µM SRCI1 for 30 min. Full-size Western blot membranes, from which the bar plots were calculated, are provided in the Supplementary. Mean values ± SD, n = 3-4 are shown. * – p < 0.05, ** – p < 0.01, *** – p < 0.001 compared to the control.

According to the data obtained in our previous studies (Petrushanko et al., 2022), Aβ_42_ induces Src kinase activation in SH-SY5Y cells. Treatment by100 nM Aβ_42_ increased APP level in cells by 75%, whereas 10 μM Src inhibitor (SRCI1) pre-treatment completely prevented this effect (Figures 2A,B). Notably, the inhibitor itself did not affect APP accumulation. Src kinase activation in these probes was evaluated as a ratio of phosphorylated at Y419 Src kinase (p-Src) to total Src kinase signals. SRCI1 decreases Src activation in cells and prevents its growth after Aβ_42_ treatment (Figures 2C,D). The obtained data show that Src kinase activation has a crucial role in the Aβ_42_-mediated gain of APP accumulation.

Earlier, we demonstrated that incubation with Aβ_42_ during 30 min declines GSH and ROS levels in SH-SY5Y cells (Petrushanko et al., 2022). This change in redox status is not associated with changes in calcium and mitochondrial potential (Supplementary Figure S2). To find out whether the observed change in redox status of cells is associated with Src kinase activation and altered APP trafficking, we added Src kinase inhibitor to the cells that were further exposed to Аβ_42_, and evaluated whether the inhibition of Src kinase affected the Aβ_42_ -induced changes in GSH (Figure 2E). The 30-min pre-treatment with 10 mM SRCI1 before exposing to 100 nM Aβ_42_ does not prevent Aβ_42_-induced changes in GSH levels, suggesting that the reduced glutathione decrease in the presence of Аβ_42_ is achieved by mechanisms that do not imply Src kinase activation. Also, it does not change the effect of Aβ_42_ on ROS level (Supplementary Figure S3). Therefore, Aβ_42_-induced increase in APP level does not depend on the redox status of cells.

### Ouabain diminishes Aβ_42_-mediated amplification of APP level

Activation of Src kinase is induced by the interaction of Aβ_42_ with Na,K-ATPase (Petrushanko et al., 2022). Earlier, we demonstrated that CTS ouabain binding to Na,K-ATPase does not affect its binding to Aβ_42_ (Adzhubei et al., 2022). Therefore, we presumed that ouabain can modulate effects caused by Аβ_42_ via Na,K-ATPase. To study the potential ability of ouabain to modulate the effect of Aβ_42_ on neuronal cells, we performed a series of experiments with 100 nM ouabain, which is equimolar to Aβ_42_. At this concentration, ouabain does not exert a toxic effect on the cells (Supplementary Figure S4).

**FIGURE 3:**
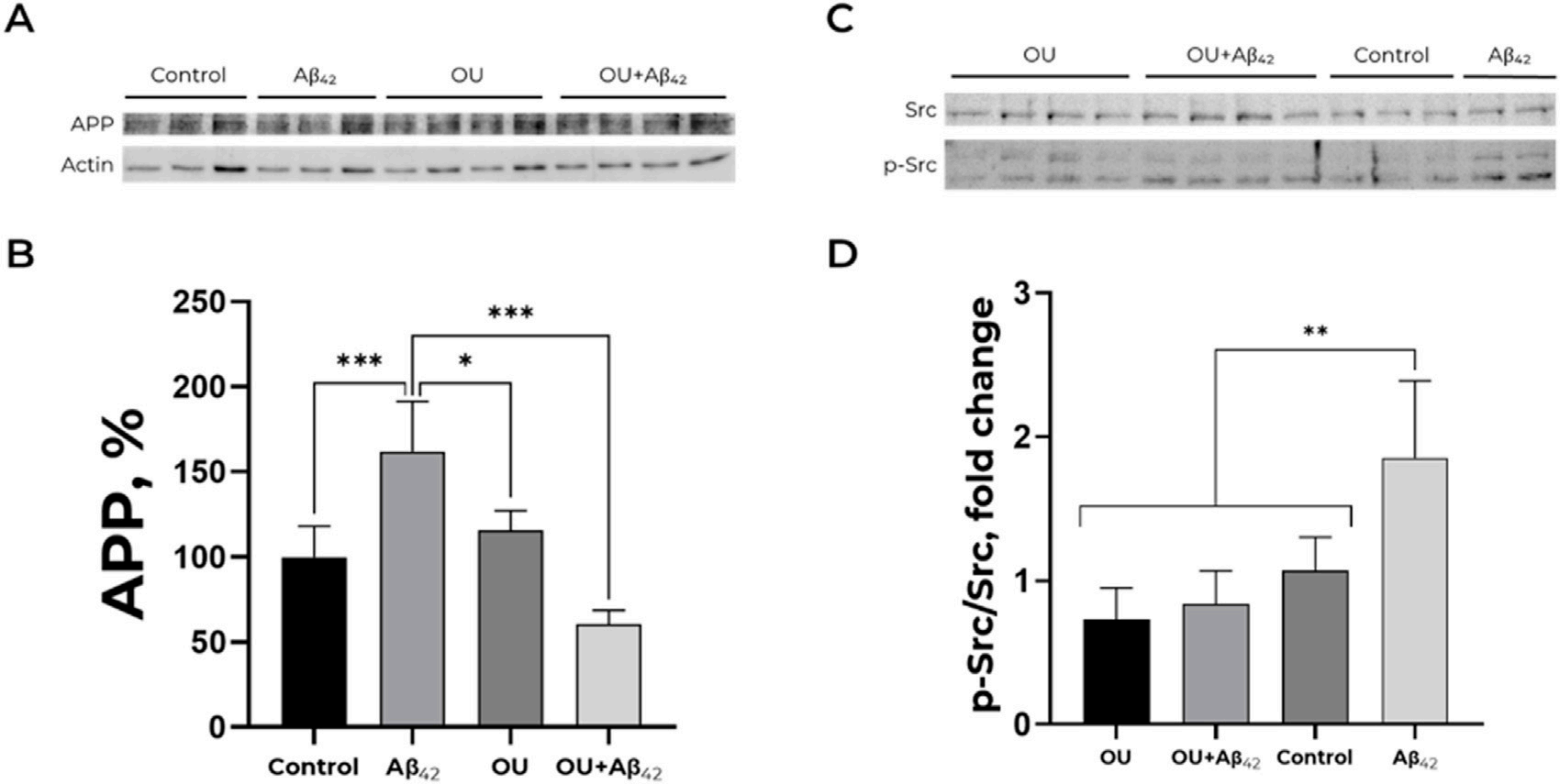
Effect of ouabain and Aβ_42_ on the APP level and Src kinase activation. **(A,B)** Impact of Aβ_42_, ouabain (OU) and Aβ_42_ after ouabain pre-treatment on APP level. The APP (∼120 kDa) to actin (∼40 kDa) ratio has been calculated for every single membrane. Full-size Western blot membranes, from which the bar plots were calculated, are provided in the Supplementary. Mean values ± SD from at least three independent experiments are shown. **(C,D)** Dependence of Src kinase activation on the presence of Aβ_42_ and/or ouabain. The ratio of phospho (Tyr)-416 Src to the total Src (∼60 kDa) has been calculated. The APP levels and the ratio of phospho (Tyr)-416 Src to the total Src were measured with Western blot in SH-SY5Y human neuroblastoma cells treated with 100 nM Aβ_42_, 100 nM ouabain or both for 30 min and normalized for control. Full-size Western blot membranes, from which the bar plots were calculated, are provided in the Supplementary. Mean values ± SD, n = 3-4 are shown. *—p < 0.05, **—p < 0.01, ***—p < 0.001 compared to the control.

Treatment of cells with 100 nM ouabain for 30 min does not alter APP accumulation (Figures 3A,B). Adding of Aβ_42_ after ouabain pre-treatment prevents the Aβ_42-_ induced increase in APP level (Figures 3A,B). Thereby, ouabain blocks the effect of Aβ_42_ on APP level and prevents the accumulation of APP in SH-SY5Y cells.

### Ouabain prevents Aβ_42_-induced Src kinase activation

The signaling cascade via Src kinase seems to be a key mechanism of the APP level amplification caused by Aβ_42_ (Figure 2). Ouabain affects Src kinase-dependent and Src-independent signaling pathways (Wu et al., 2013; Shin et al., 2015; Liu and Xie, 2010). That is why we found it important to define the impact of ouabain on Src kinase activation.

30-min incubation of SH-SY5Y cells with 100 nM ouabain does not induce Src kinase activation. Moreover, ouabain prevents Src activation by Aβ_42_ (Figures 3C,D). These data are in a good agreement with the results described above (Figures 3A,B).

## Discussion

It has been previously suggested that altered trafficking of APP in aging neurons and its accumulation in neurites with age (Burrinha and Guimas Almeida, 2022), accompanied by its intensified processing (Burrinha et al., 2021), may be responsible for the observed age-dependent increase in brain Aβ levels (Marks et al., 2017; Koinuma et al., 2021; Welikovitch et al., 2018). Moreover, APP accumulation in neurites has been described in animal models of AD (Walton and Wang, 2009). Still, it was unclear whether a feedback loop between Aβ and its precursor protein exists and whether increasing levels of Aβ can influence APP protein level and trafficking.

In this study, we found that Aβ_42_ stimulates the increase in APP level and its accumulation in neurites in human neuroblastoma SH-SY5Y cells. As local accumulation of APP sets off its amyloidogenic procession, our findings suggest the existence of a positive feedback loop via Src-kinase activation that enhances the Aβ formation. This observation suggests a number of implications regarding both the Aβ accumulation itself and the dysregulation of APP-mediated signaling cascades. First of all, pre- and postsynaptic secretion of Aβ (Niederst et al., 2015) can lead to its accumulation, oligomerization and impaired synaptic plasticity (Perdigão et al., 2020). The positive feedback loop between Aβ and APP may serve as a signal amplifier in active synapses and increase the risk of Aβ aggregation in synapses that overproduced it. In turn, it is known that, being accumulated in neuronal terminals, APP affects vesicle coupling to kinesin-I, reducing axonal transport (Stokin et al., 2005). Thus, the Aβ-induced accumulation of APP possibly reduces the efficiency of kinesin-I-mediated axonal transport.

It is important to denote the pattern of Aβ_42_-induced APP accumulation. According to our data, the total APP content in cells is increased after 30 min of Aβ_42_ exposure, followed by a steady relocation to neurites. The increase of APP level in neurites develops over time and indicates alterations in APP trafficking.

**FIGURE 4:**
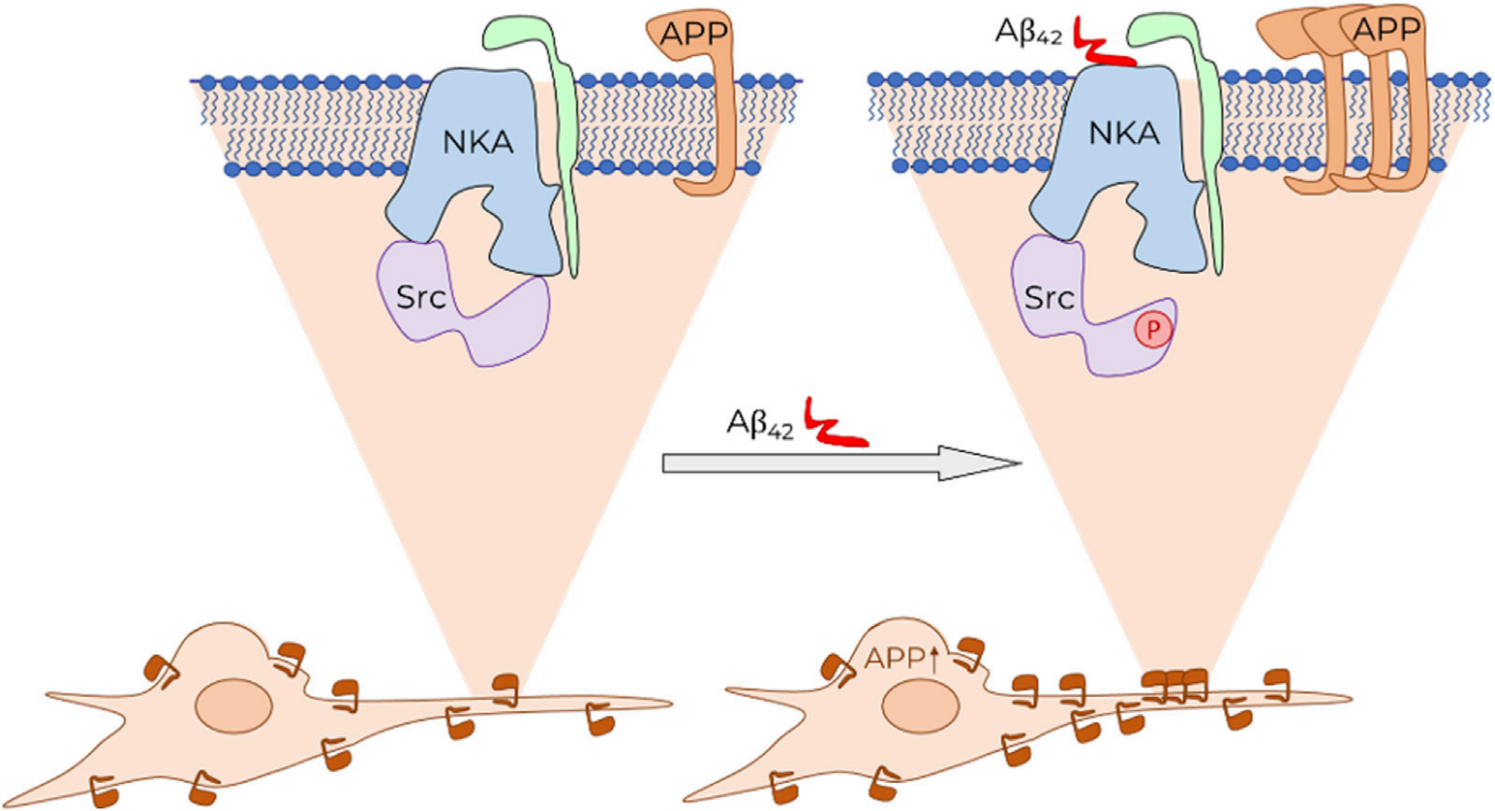
Schematic representation of Aβ effect on APP level in cells and its trafficking. Binding of Aβ (shown in red) to Na,K-ATPase (NKA, α-subunit shown in blue, β-subunit shown in green) leads to the activation of Na,K-ATPase-associated Src kinase (Src, shown in purple). This initiates a signaling cascade, which results in an increase in the total APP level in the cell. Consequently, the accumulation of APP is observed on the cytoplasmic membrane of neurites.

Earlier, we demonstrated that incubation of SH-SY5Y cells with 100 nM Aβ_42_ results in rapid (within 30 min) activation of Na,K-ATPase-associated Src kinase via Aβ_42_ binding to Na,K-ATPase (Petrushanko et al., 2022) and does not change Na,K-ATPase activity. We hypothesized that Src kinase may be involved in the regulation of APP trafficking (Petrushanko et al., 2022). This hypothesis was confirmed using a Src kinase inhibitor, which completely prevented Аβ-mediated upregulation of APP (Figure 2A). Thus, we assert the following sequence of events that form the positive feedback loop (Figure 4). First, binding of Аβ to Na,K-ATPase activates the Na,K-ATPase-associated Src kinase. In turn, Src kinase causes an increase in APP levels and its transport into neurites. It is likely that self-amplification of Aβ synthesis by the proposed mechanism serves as one of the key events in the pathogenesis of Alzheimer’s disease and explains the exponential pattern of its development (Dickson, 1997).

It should also be noted that, apart from a positive one, a negative feedback loop is also possible. In particular, Src kinase can activate phospholipase C, enhancing α-secretase activity involved in APP cleavage via the non-amyloidogenic pathway (Pimenova et al., 2014). Thus, it is possible that activation of Src kinase by Aβ enhances APP level in cells, simultaneously increasing the contribution of the non-amyloid pathway to its proteolysis and preventing beta-amyloid from further excessive formation. On the other hand, the effect of Src kinase on APP may also turn out to be indirect. Namely, the phosphorylation of Src kinase by trafficking adapter Mint leads to APP accumulation in the trans-Golgi network, which impairs its transport through dendrites to synaptic terminals (Dunning et al., 2016). However, in our case, conversely, the level of APP in neurites is increased (Figure 1), hence another regulatory pathway can be assumed.

One could also suppose that one of the possible indirect ways in which Src kinase affects APP is via alterations of the redox status of cells. In particular, this could be caused by Аβ-mediated reduction in ROS and reduced glutathione (GSH) levels that occurs after 30 min of incubation in SH-SY5Y cells, as we have shown in (Petrushanko et al., 2022). However, inhibition of Src kinase exerted no effect on the Аβ-mediated reduction of GSH, which proves the independence of these effects (Figure 2E).

Since Src kinase activation is mediated by the binding of Aβ_42_ to Na,K-ATPase, which also acts as the receptor for cardiotonic steroids, we hypothesized that ouabain may influence the positive feedback loop we found. This hypothesis was confirmed: at a concentration of 100 nM, ouabain prevents Aβ_42_-induced activation of Src kinase and increase in APP levels, which makes it a promising CTS for preventing Aβ_42_-induced APP growth.

Earlier, the possibility of using CTS as part of the complex therapy of Alzheimer’s disease has been evaluated (Nguyen et al., 2023; Wang et al., 2024; Song et al., 2019; Erdogan et al., 2022). It was demonstrated that ouabain and digoxin, another cardiotonic steroid, can prevent cytotoxic effects in neurons due to inhibition of tau protein synthesis. This process is mediated by miR-132, one of the key neuroprotective miRNAs that is suppressed in Alzheimer’s disease (Nguyen et al., 2023). Moreover, ouabain is able to reduce microglial neuroinflammation in murine models of Alzheimer’s disease (Wang et al., 2024). Although the data obtained in mice are difficult to extrapolate to humans due to the presence of a ouabain-resistant Na,K-ATPase isoform in rodents (Poluektov et al., 2025), our data support the idea of considering ouabain as a possible addition to Alzheimer’s disease therapy. It is ouabain that can offset the effect of Aβ, thereby preventing the development of a feedback loop between APP and Aβ and, as a result, reducing the dramatic increase in beta-amyloid in Alzheimer’s disease. Our findings demonstrate the possibility of diminished beta-amyloid-induced effects not only by regulating the activity of microglia (Wang et al., 2024), but also by direct action of ouabain on neuronal cells. Remarkably, 100 nM ouabain does not inhibit Na,K-ATPase and does not affect the levels of sodium and potassium in SH-SY5Y cells after 30 min of incubation (Kulikov et al., 2007). Thus, observed effect of ouabain on Src kinase activation and beta-amyloid-induced changes in APP levels is not associated with Na,K-ATPase inhibition. Further, since ouabain binding to Na,K-ATPase does not prevent amyloid binding (Adzhubei et al., 2022), it can be concluded that ouabain and Aβ are likely not to share the same binding site. One can suggest that ouabain prevents the signaling effects of beta-amyloid that are mediated by conformational changes of Na,K-ATPase (Klimanova et al., 2015).

Notably, ouabain is detectable in cerebrospinal fluid at higher concentrations than in plasma (Dvela et al., 2012). Endogenous ouabain is now recognized not only as a centrally acting hormone but also as a paracrine neurohormone locally produced in the CNS, likely by the hypothalamus (Weidemann et al., 2004; Leenen et al., 2017). The levels of endogenous CTSs may vary under pathological conditions (Orlov et al., 2021). In a murine model of AD, marinobufagenin levels are shown to be decreased. Administration of exogenous marinobufagenin has been demonstrated to reduce neuroinflammation and lower IL-6 levels (Fedorova et al., 2020). Despite the limited knowledge regarding CTS levels in AD patients, we hypothesize a decline in endogenous CTS levels in AD. This assumption is supported by previous observations indicating that the hypothalamus, a primary source of endogenous CTSs in the brain, is suppressed in AD. Particularly, levels of sex hormones were decreased in murine AD models (Qi et al., 2024). Our data on ouabain modulation of beta-amyloid-induced alterations in APP levels and trafficking suggest its key regulatory role in beta-amyloid signaling. In this context, restoring physiological levels of CTSs could potentially inhibit AD progression. The administration of exogenous ouabain could restore ouabain levels to physiologically normal without inhibiting Na,K-ATPase, but rather slowing the accelerated formation of APP.

To sum up, the data suggest that Aβ activates Src kinase by binding to Na,K-ATPase, causing an increase in the APP level and its relocation to neurites in SH-SY5Y cells. This can lead to an even greater increase in beta-amyloid levels due to APP processing and a feedback loop. Specific Na,K-ATPase ligand, cardiotonic steroid ouabain, prevents impact of beta-amyloid on Src kinase and APP, which makes it a potential therapeutics against Alzheimer’s disease.

## Data Availability

The original contributions presented in the study are included in the article/Supplementary Material, further inquiries can be directed to the corresponding authors.
