# Supplementary material for: Beta-amyloid influences the content and trafficking of beta-amyloid precursor protein via Na,K-ATPase-Src kinase positive feedback loop
Source: Front Pharmacol. 2025 Sep 24;16:1665715. doi: 10.3389/fphar.2025.1665715 (PMC12504301; doi:10.3389/fphar.2025.1665715)
Supplement: Supplementary file 1 [file DataSheet1.pdf]

Supplementary Material

1.1 Supplementary Figures

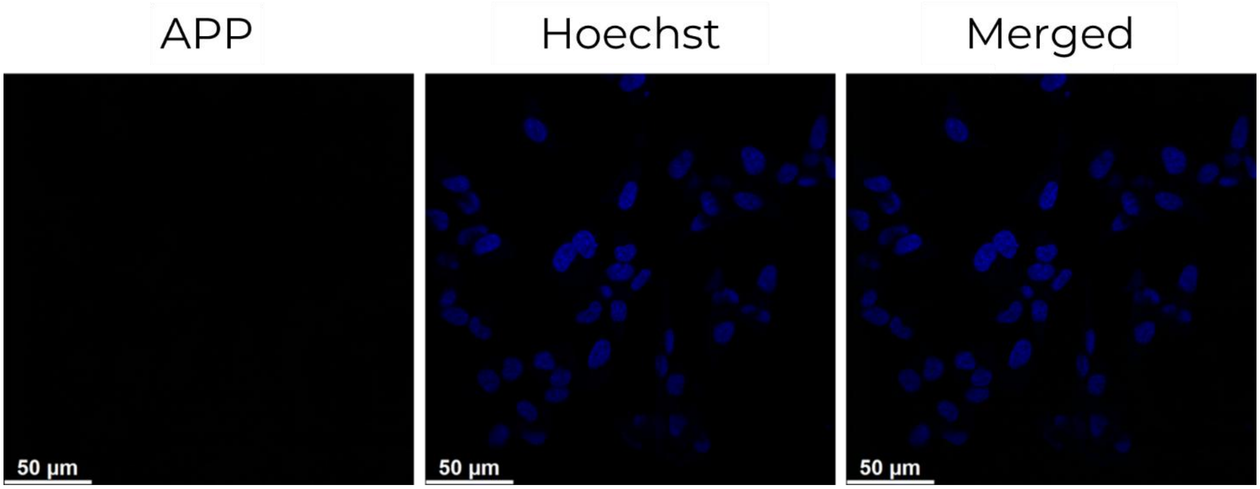

Supplementary Figure 1. Confocal images of the control specimens without treatment with primary antibodies to APP. Staining of nuclei with NucBlue (Hoechst 33342) dye is given in blue.

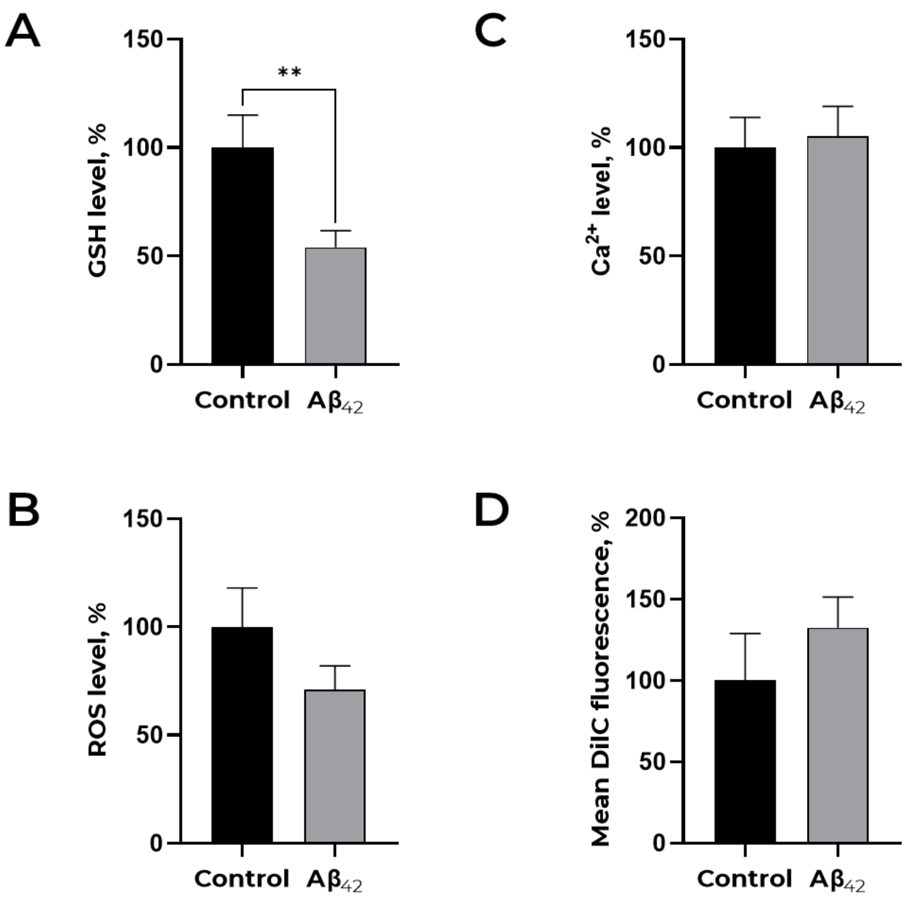

Supplementary Figure 2. Effect of Aβ$_{42}$ on intracellular redox-parameters, calcium level and mitochondrial potential in SH-SY5Y human neuroblastoma cells. **(A)** Reduced glutathione level in non-treated and Aβ$_{42}$ treated cells. **(B)** An impact of Aβ$_{42}$ on intracellular reactive oxygen species level (ROS). **(C)** Cytosolic Ca^{2+} level under Aβ$_{42}$ treatment. **(D)** DilC$_1$(5) fluorescence intensity which is corresponding the magnitude of mitochondrial potential in non-treated and Aβ$_{42}$ treated cells. The cells were harvested and stained with fluorescent dyes: monobromobimane for GSH measurements, fluo-4 for Ca^{2+} level measurements, dyhydrorhodamine 123 for ROS level measurements, DilC$_1$(5) for mitochondrial membrane potential measurements and incubated with 100 nM Aβ$_{42}$ for 30 minutes. All parameters were normalized for control. Mean values ± SD, n=4 are shown. ** – $p < 0.01$ compared to the control.

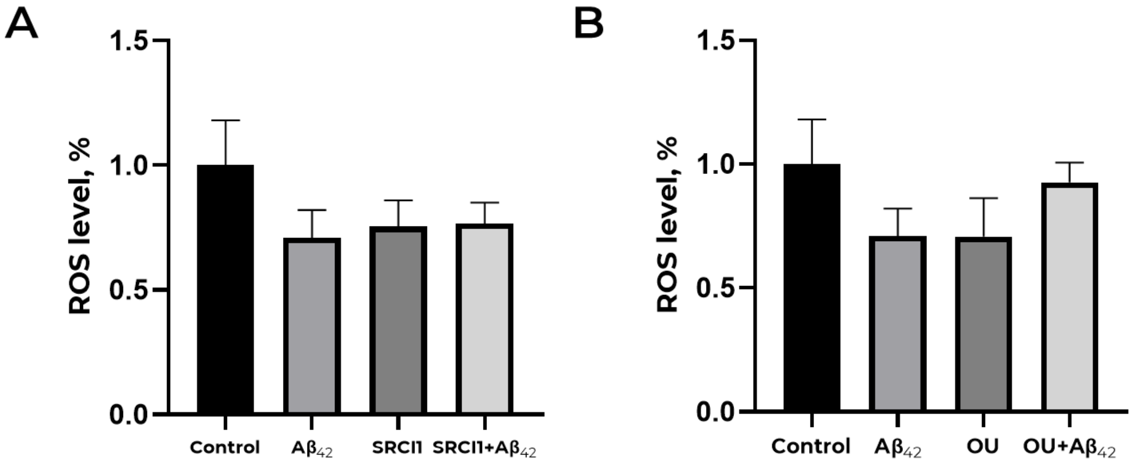

Supplementary Figure 3. Evaluation of the effect of Aβ42 on the level of reactive oxygen species (**A**) ROS levels in a presence of Aβ42, Src kinase inhibitor 1 and both. (**B**) An impact of Aβ42 and ouabain (OU) on intracellular ROS level. The SH-SY5Y human neuroblastoma cells were harvested and stained with dyhydrorhodamine 123 for ROS level measurements and incubated with 100 nM Aβ42 and, if required, 10 μM SRCI1 or 100 nM ouabain for 30 minutes. All parameters were normalized for control. Mean values ± SD, n=4 are shown.

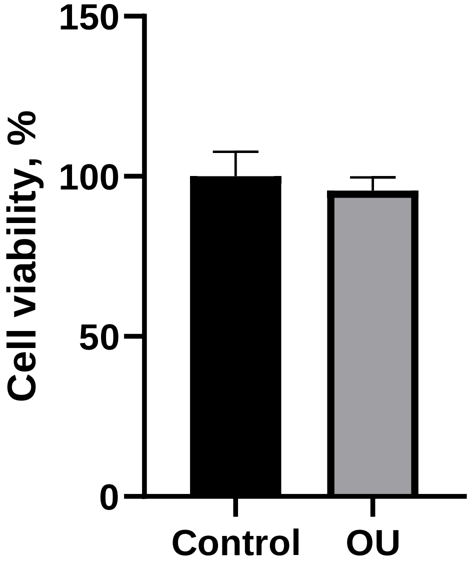

Supplementary Figure 4. WST cell viability tests results after the treatment SH-SY5Y neuroblastoma cells with 100 nM ouabain (OU) and without ouabain (Control) for 24 hours. Mean values ± SD, n=4 are shown.

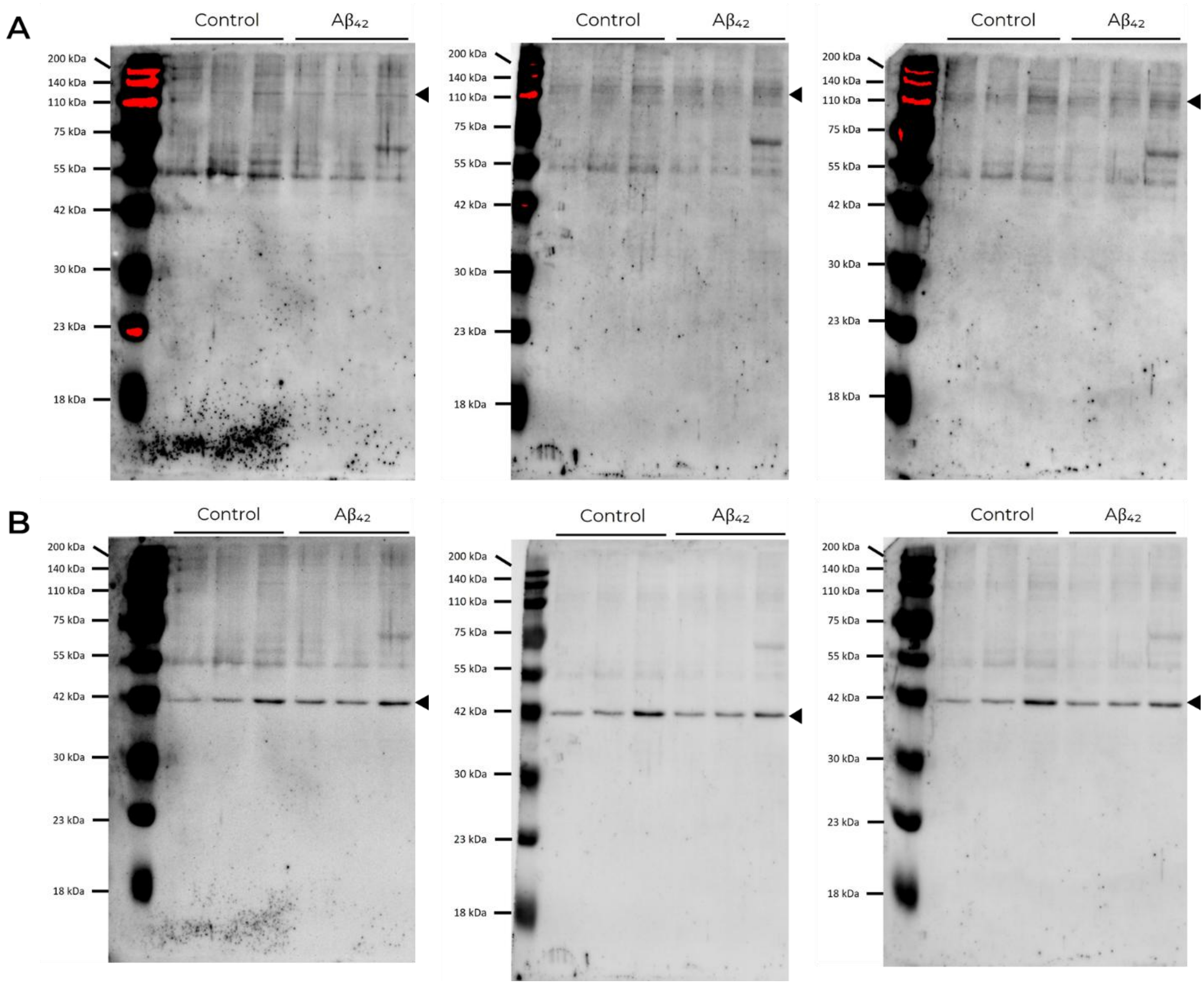

Supplementary Figure 5. Full-size Western-blot membranes stained with the primary antibodies to APP (A) and actin (B). The black arrows show bands which were analyzed in ImageLab.

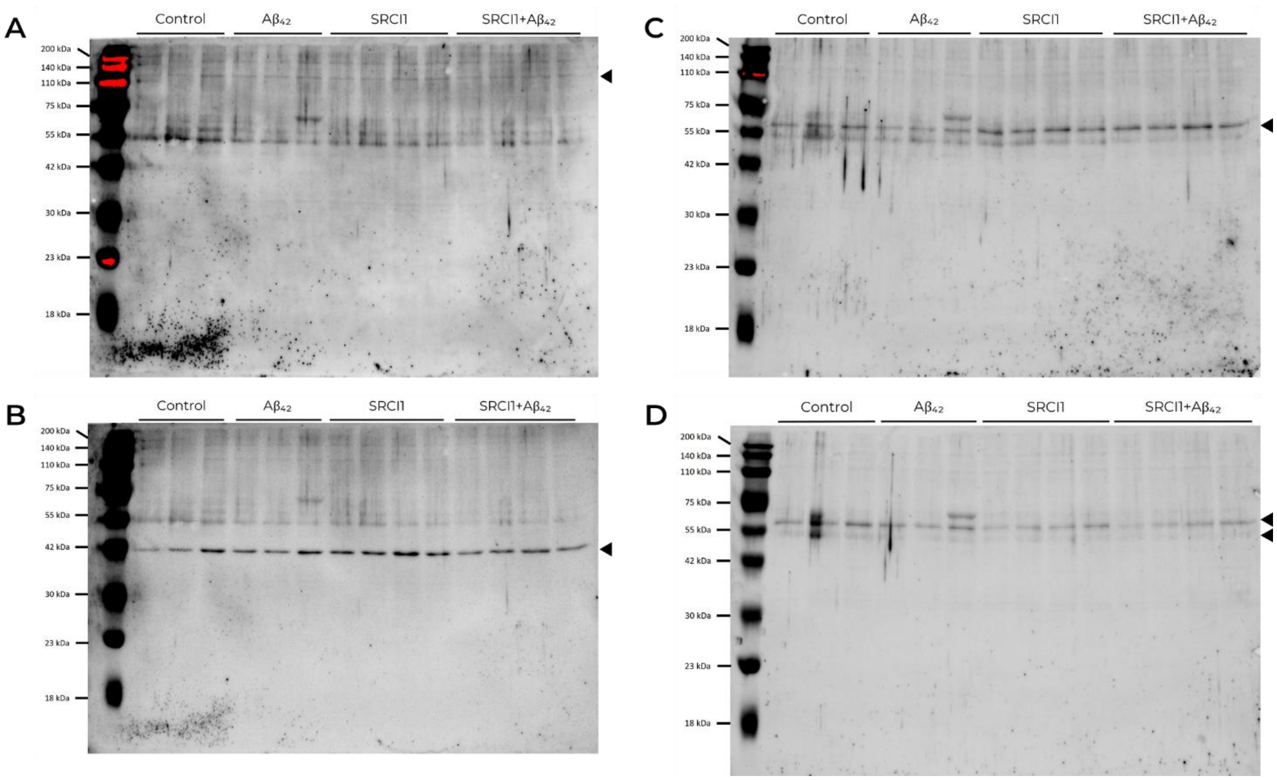

Supplementary Figure 6. Full-size Western-blot membranes with the samples which were treated with 10 μM Src kinase inhibitor (SRCI1). Membranes were stained with the primary antibodies to APP (A), actin (B), Src (C) and p-Src (D). The black arrows show bands which were analyzed in ImageLab.

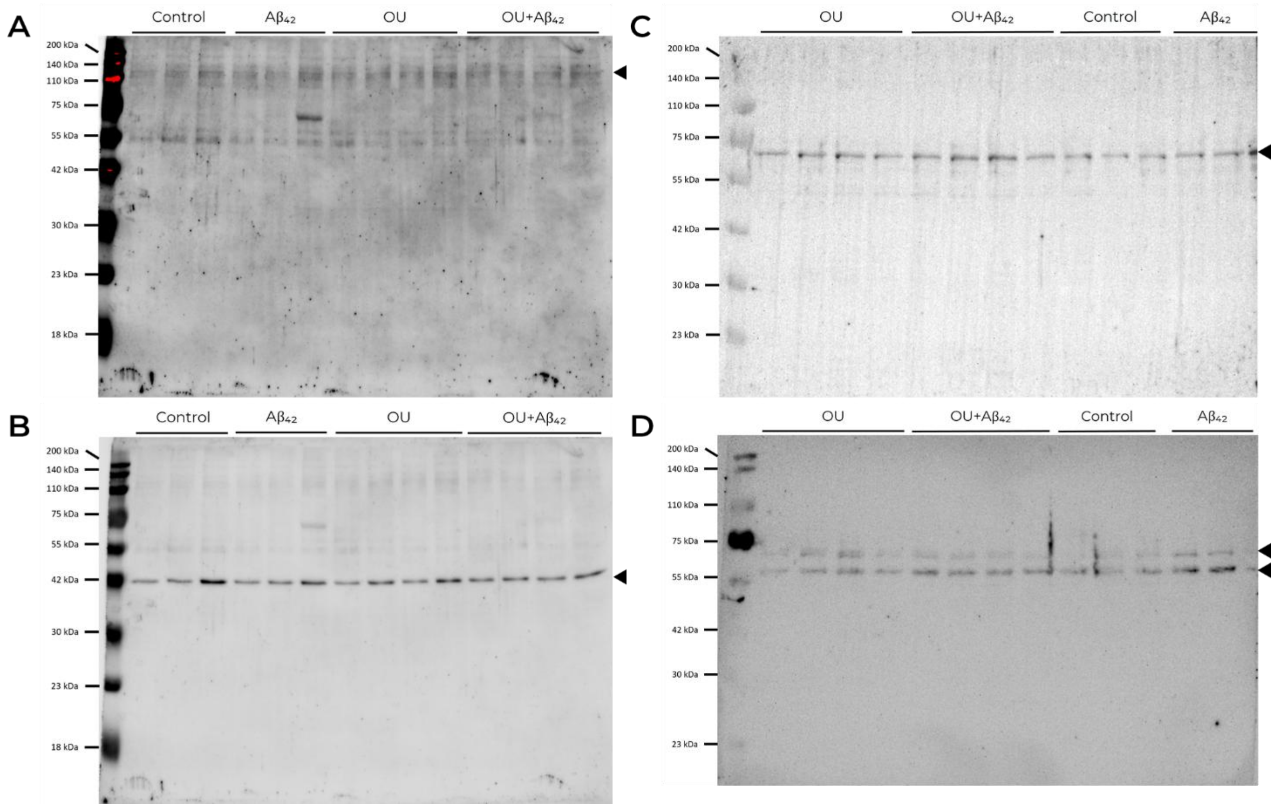

Supplementary Figure 7. Full-size Western-blot membranes with the samples which were treated with 100 nM ouabain (OU). Membranes were stained with the primary antibodies to APP (A), actin (B), Src (C) and p-Src (D). The black arrows show bands which were analyzed in ImageLab.
